# Phytotoxic Metabolites Produced by Legume-Associated *Ascochyta* and Its Related Genera in the Dothideomycetes

**DOI:** 10.3390/toxins11110627

**Published:** 2019-10-29

**Authors:** Wonyong Kim, Weidong Chen

**Affiliations:** 1 Department of Plant Pathology, Washington State University, Pullman, WA 99164, USA; apbiomol@gmail.com; 2 Korean Lichen Research Institute, Sunchon National University, Suncheon, Jeonnam 57922, Korea; 3 USDA-ARS Grain Legume Genetics and Physiology Research Unit, Pullman, WA 99164, USA

**Keywords:** phytotoxin, *Ascochyta*, *Phoma*, *Didymella*, secondary metabolites, gene cluster, legumes

## Abstract

Phytotoxins, secondary metabolites toxic to plants and produced by fungi, are believed to play an important role in disease development by targeting host cellular machineries and/or interfering with host immune responses. The Ascochyta blight diseases on different legume plants are caused by *Ascochyta* and related taxa, such as *Phoma*. The causal agents of the Ascochyta blight are often associated with specific legume plants, showing a relatively narrow host range. The legume-associated *Ascochyta* and *Phoma* are known to produce a diverse array of polyketide-derived secondary metabolites, many of which exhibited significant phytotoxicity and have been claimed as virulence or pathogenicity factors. In this article, we reviewed the current state of knowledge on the diversity and biological activities of the phytotoxic compounds produced by *Ascochyta* and *Phoma* species. Also, we touched on the secondary metabolite biosynthesis gene clusters identified thus far and discussed the role of metabolites in the fungal biology.

## 1. Introduction

The Dothideomycetes is the largest class of fungi within the phylum Ascomycota and represents diverse forms of life, such as saprobes, endophytes, mycorrhizae, marine fungi, lichenized fungi and pathogens [1]. Members of the Dothideomycetes include some notorious pathogens with quarantine status, which cause significant yield losses in our major crops [2]. The anamorphic genera *Ascochyta* and *Phoma* in the Dothideomycetes are polyphyletic: A majority of the members (approximately 70%) are found in the family Didymellaceae, but others are found in other taxa in the order Pleosporales [3,4]. However, recent molecular phylogeny indicated that *Ascochyta* and *Phoma* should be restricted to the Didymellaceae [4]. Species of *Ascochyta* and *Phoma* share morphological and physiological features and are responsible for diseases on many cool season food legumes, often referred to as Ascochyta blight [5,6]. The diseases are manifested through necrotic lesions on above-ground tissues including leaves, stems, and pods. A histological study showed that cells were damaged and disintegrated even before direct fungal contact, seemingly attributing necrosis to secreted phytotoxic compounds [7].

The Ascochyta blight diseases are caused by different *Ascochyta* species in a host-specific manner in many instances: *Ascochyta fabae* Speg., *A. lentis* Vassiljevsky, *A. pisi* Lib., *A. rabiei* (Pass) Labr., and *A. viciae-villosae* Ondrej are pathogens of faba bean (*Vicia faba* L.), lentil (*Lens culinaris* Medik.), pea (*Pisum sativum* L.), chickpea (*Cicer arietinum* L.), and hairy vetch (*Vicia villosa* Roth), respectively [8,9,10,11]. Recently, Italian isolates of *A. lentis* var. *lathry* were shown to be pathogenic to grasspea (*Lathyrus sativus* L.), but less pathogenic to lentil, suggesting further host specialization in *A. lentis* and *A. lentis* var. *lathry*, the two biologically compatible species [12]. These host-specific *Ascochyta* spp. have been linked all to the *Didymella* teleomorphs [10,13]. The new teleomorphe *Peyronellaea* was recently assigned to *A. pinodes* L.K.Jones (formerly *Didymella pinodes*) [4]. *Ascochyta pinodes* exhibited a broader host range: The fungus was able to develop disease symptoms on 19 different legumes, although it was most aggressive on pea, the primary host [14]. *Phoma medicaginis* Malbr. and Roum. (syn. *A. medicaginicola*) also had multi hosts infecting alfalfa, lentil and chickpea [15]. 

*Ascochyta* and *Phoma* are distinguished based on conidial morphology in the Saccardoan system: *Ascochyta* spp. produce two-celled conidia, while *Phoma* spp. produce one celled conidia [3]. However, the cell number of conidia is not an absolute criterion for distinguishing the two genera, because one-celled conidia are also produced by *Asochyta* species. The legume-associated *Ascochyta* and *Phoma* spp. are known to produce polyketide-derived secondary metabolites (SMs), many of which display significant toxicity to plants. Despite the limited morphological characters to distinguish these legume-associated *Ascochyta* and *Phoma* spp., they can be classified, to some extent, solely with chemical profiles of the fungal culture extracts, suggesting they have shaped unique metabolic profiles during speciation [16]. In this respect, the legume-associated *Ascochyta* and *Phoma* spp. can be a good model system for studying host specificity and chemical evolution within the closely related species.

Species belong to the Dothideomycetes, such as plant pathogenic *Alternaria* and *Cochliobolus* spp., are known to produce polyketide-derived SMs that are toxic only to the host plants and that play key roles in pathogenicity (e.g., ACR-toxin and T-toxin) [17,18,19,20,21]. However, the phytotoxic compounds produced by the legume-associated *Ascochyta* and *Phoma* are often toxic to a range of plants in addition to the respective host plants and their roles in pathogenicity are uncertain. Here, we review these phytotoxic SMs produced by the legume-associated *Ascochyta* and *Phoma*, focusing on their diversity, biological activities and biosynthesis gene clusters.

## 2. Biological Activities and the Modes of Action

### 2.1. Ascochitine and Its Derivatives

**Figure 1:**
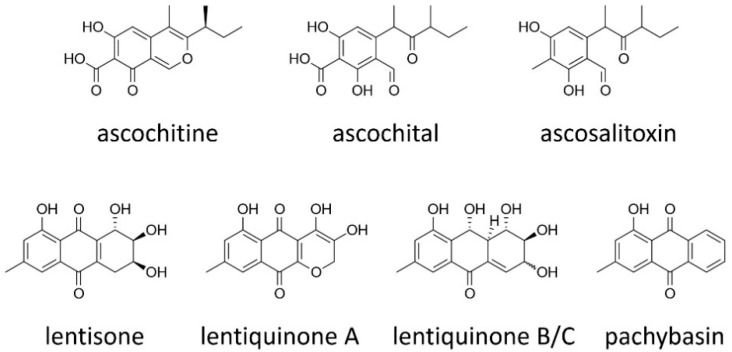
Asochitine and anthraquinones produced by the legume-associated *Ascochyta* and *Phoma* species. Lentiquinones B and C are epimers.

Ascochitine, an *o*-quinone methide, was first discovered in culture extracts of *A. pisi* [22] and later in *A. fabae* [23] (Figure 1). Recently, ascochitine was found in culture extracts of many wild vetch-infecting *Ascochyta* and *Ascochyta*-like species including *A. viciae-villosae* [16]. The widespread distribution of ascochitine production indicates its ancient origin in these related taxa. Ascochitine production is not restricted to the legume-associated *Ascochyta* species. A pea pathogen *P. koolunga* and other non-legume pathogens *P. clematidina* (syn. *Calophoma clematidina*) and *A. hyalospora* (syn. *Pleospora chenopodii*) are also ascochitine producers [24,25]. In addition, ascochitine has been isolated from two endophytic fungi *A. salicorniae* (syn. *Stagonosporopsis salicorniae*) and *Anteaglonium* sp. [26,27]. These diverse fungi producing ascochitine all belong to the order Pleosporales in Dothideomycetes, suggesting ascochitine production is ancestral to the legume-associated *Ascochyta* and *Phoma* species.

The association of ascochitine producers with plants and an alga (*Ulva* sp.) suggests ascochitine possibly plays a role in the infection and/or colonization of these green organisms. The phytotoxicity of ascochitine was examined by applying purified ascochitine directly to leaf discs of different *Clematis* cultivars. Interestingly, ascochitine caused electrolyte leakage from leaf discs of cultivars susceptible to *P. clematidina*. However, cultivars resistant to the fungus were largely insensitive to ascochitine [24]. Ascochitine was recovered from plant tissues colonized by *P. clematidina*, indicating the production of ascochitine during plant infection [24]. Ascochitine was originally reported to be a selective antifungal agent, inhibiting the growth of certain fungi [28,29]. Some insensitive fungi were able to metabolize ascochitine, and the metabolizing ability was proportional to the tolerance level against ascochitine [29]. Ascochitine is also known to inhibit the enzymatic activity of a bacterial tyrosine phosphatase [27].

Ascosalitoxin and ascochital are precursors and end products of the ascochitine biosynthetic pathway, respectively [27] (Figure 1). Ascosalitoxin exhibited phytotoxicity to legumes and tomato seedlings [30], while ascochital displayed antimicrobial activities [27,31].

### 2.2. Anthraquinones

Anthraquinones are produced by many different fungi and plants, but the pathways of their biosynthesis are different: Fungal anthraquinones are primarily synthesized via the polyketide pathway, while plant anthraquinones are synthesized via either the polyketide or the shikimate pathway [32,33]. Many anthraquinones that have been isolated from dothideomycetes fungi have been reported as phytotoxins and play diverse roles, such as UV protection, antibiosis, signaling, and redox reactions [34,35,36,37,38,39]. Nevertheless, their roles in pathogenicity have not yet been clearly demonstrated. Anthraquinones are among the largest groups of natural products, but only *A. lentis* is known to produce anthraquinones in the legume-associated *Ascochyta* species [40,41]. Although *A. lentis* is phylogenetically closely related to ascochitine producers, such as *A. fabae*, *A. pisi* and *A. viciae-villosae* [9], ascochitine has not been found in culture extracts of *A. lentis* isolates [16], suggesting the metabolic profiles of *A. lentis* have been diversified from the related species. Among diverse anthraquinones produced by *A. lentis*, lentiquinones, lentisone and pachybasin exhibited significant biological activities (Figure 1).

Lentisone exhibited strong phytotoxicity, causing necrotic lesions when externally applied to leaf disks of different legumes [40], and was active against the Gram-positive bacterium *Bacillus subtilis*, causing an inhibition zone [41]. Lentiquinones also caused necrosis on plant leaves, but on average to a lesser extent than lentisone [41]. Lentiquinone C was most active against *B. subtilis*, while lentiquinone A was most phytotoxic, suggesting the difference in the functional groups of the compounds is important for specificity to different organisms [41]. Pachybasin did not exhibit any phytotoxicity nor antimicrobial activities [40,41]. Instead, pachybasin and another anthraquinone emodin are implicated in the mycoparasitic activity of *Trichoderma harzianum* [39]. However, the mode of action, cellular targets and the responsible biosynthetic genes for these bioactive anthraquinones remain to be characterized.

### 2.3. Macrolides

**Figure 2:**
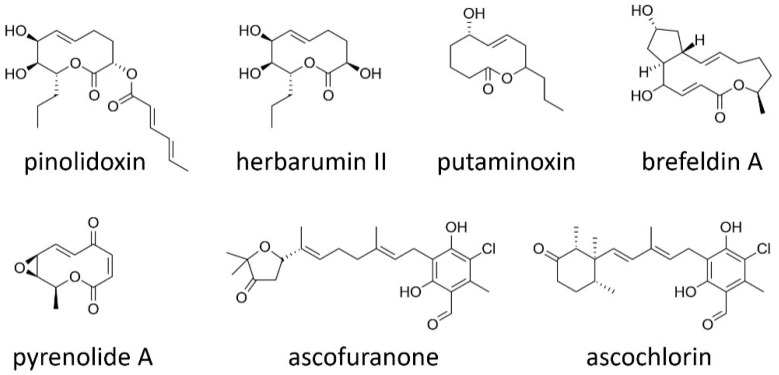
Macrolides and meroterpenoids produced by the legume-associated *Ascochyta* and *Phoma* species.

Pinolidoxin is a 10-membered macrolide with a medium-sized lactone ring, produced by *A. pinodes* and *A. pinodella* [16,42]. In addition, *A. pinodes* produces structurally related 10-membered macrolides, such as herbarumin II, pinolide, and pinolidoxin derivatives (Figure 2)—of which, pinolidoxin exhibited the strongest phytotoxicity [43]. Another 10-membered macrolide putaminoxin is produced by a plant pathogenic fungus *P. putaminum* [44]. Both pinolidoxin and putaminoxin are potent inhibitors of phenylalanine ammonia lyase that catalyzes the first step of phenylpropanoid pathway [45]. The structure–activity relationship study with pinolidoxin, putaminoxin and their natural or synthetic derivatives revealed that the two hydroxyl groups and the unmodified propyl side chain at the lactone ring are crucial for the observed phytotoxicity [46]. The phenylpropanoid pathway of higher plants comprises the two main branches leading to the biosynthesis of lignin and flavonoids including many phytoalexins that play roles in defense against microbial and animal parasites. Pinolidoxin was detected using MALDI imaging mass spectrometry in hyphae on the growing front of the *A. pinodies* colony [47]. The available evidence of in situ localization, the broad spectrum of phytotoxicity and the mode of action suggest that pinolidoxin plays a role during hyphal invasion likely by modulating plant defense responses.

*Phome medicaginis* (syn. *A. medicaginicola*), a pathogen of *Medicago* spp, produces the 13-membered macrolide brefeldin A (Figure 2). Brefeldin A was originally identified in *Eupenicillium brefeldianum* and in *Alternaria carthami*, a pathogen of safflower (*Carthamus tinctorius* L.) [48,49]. In vitro phytotoxicity tests showed that brefeldin A caused necrotic lesions on safflower leaves when applied externally [48]. Brefeldin A is known to block protein transport from the endoplasmic reticulum to the Golgi apparatus, thus having been used in studies on intracellular membrane trafficking systems [50,51]. Despite the apparent phytotoxicity, brefeldin A was not detected in infected plant tissues, although it was produced in the culture of *P. medicaginis* [52]. Some bioactive macrolides are also found in culture extracts of *Ascochyta* and *Phoma* spp. associated with non-legume hosts (Figure 2). *Phoma herbarum* produces herbarumin congeners that displayed significant phytotoxicity and antimicrobial activities [53,54,55]. *Ascochyta hyalospora* produces an antifungal macrolide pyrenolide A [25].

### 2.4. Meroterpenoids

Ascofuranone and ascochlorin of polyketide–terpene hybrid origin were identified in culture extracts of *A. viciae* that causes diseases on vetches (*Vicia* spp.) decades ago [56,57] (Figure 2). However, the ascofuranone-producing fungal isolate was recently reexamined and identified as *Acremonium sclerotigenum* [58]. This species identification is consistent with previous reports on the occurrence of natural analogs of ascofuranone in several fungal taxa related to *Acremonium*, such as *Colletotrichum*, *Cylindrocladium*, *Fusarium* and *Verticillium* in Sordariomycetes [59,60,61,62,63]. Thus, the production of ascofuranone and ascochlorin by *A. viciae* has to be carefully re-examined. Ascofuranone and ascochlorin have gained great attention due to their potential as chemotherapeutic agents and antitrypanosomal drugs [64,65]. The two compounds are structural analogs of ubiquinol, an essential component of the respiratory chain for ATP synthesis, and thus inhibit the enzymatic activities of protozoan alternate oxidase by acting at the ubiquinol binding site [66,67]. Ascochlorin was reported to inhibit the respiratory chain of the ascomycetes yeast *Pichia anomala* by targeting the mitochondrial cytochrome *bc1* complex, also known as coenzyme Q [68].

### 2.5. Solanapyrones

**Figure 3:**
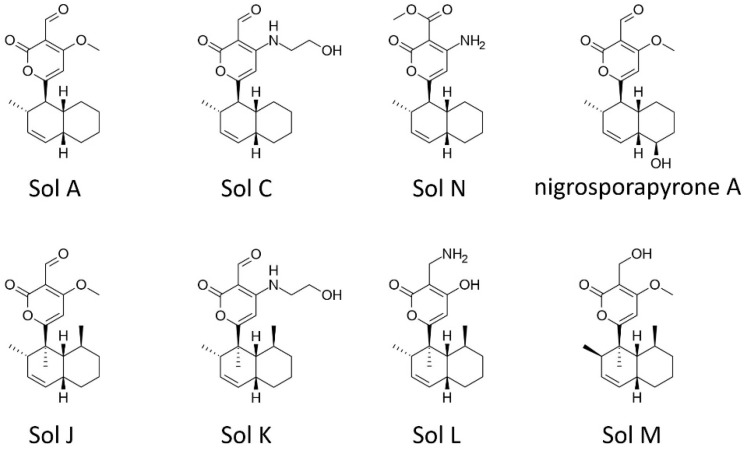
Solanapyrone congeners produced by *Ascochyta rabiei* and natural analogs found in other fungal extracts. Sol: Solanapyrone.

Solanapyrones, α-pyrones with a decalin ring, were originally identified in the culture extract of *Alternaria solani*, the causal agent of early blight of potato (*Solanum tuberosum* L.) [69], and later in *A. rabiei* [70] (Figure 3). All *A. rabiei* strains examined to date produce at least one of the solanapyrone congeners, and the disease-causing abilities of *A. rabiei* isolates on chickpea plants has been correlated with their ability to produce solanapyrones [71,72]. When purified solanapyrone A was directly applied to cuttings of chickpea shoots, it caused loss of turgor within the shoot cuts [73]. However, solanapyrone A cannot be recovered from chickpea tissues after the wilting symptom develops, and the authors suggested that glutathione-*S*-transferase in chickpea tissues may detoxify solanapyrone A by covalently linking glutathione to solanapyrone A [73]. Also, purified solanapyrone A caused necrotic lesions on leaflets of different legume plants and inhibited primary root growth of *Arabidopsis thaliana*, but induced lateral root branching [74].

Many solanapyrone analogs have been found in fungi occupying different ecological niches and exhibited broad spectrum antimicrobial activities (Figure 3) [75,76,77,78,79]. Solanapyrone A exhibited antibacterial activities to *Bacillus subtilis* and *Micrococcus tetragenus* as strong as the antibiotic ampicillin, whereas solanapyrone C appeared to be more selective, affecting only *B. megaterium* among the several tested bacteria [79]. Interestingly, solanapyrone C also showed an algistatic effect to a unicellular algal species [75]. Purified solanapyrone A inhibits the growth of saprobic fungi isolated from chickpea debris [47]. Solanapyrones J and K exhibited antimicrobial activities to *Candida albicians* and *Staphylococcus aureus* (Gram-positive), but did not affect *Escherichia coli* (Gram-negative) [76]. Solanapyrones L and M differ in functional groups on the pyrone ring from those in solanapyrones J and K [76]. These natural analogs give insight into the relationship between chemical structure and activity, as solanapyrones L and M did not exhibit any significant activities [76]. Solanapyrone N was effective in suppressing the growth of *Botrytis cinerea* and *Penicillium islandicum* among seven tested fungi [78]. Nigrosporapyrone A exhibited a moderate toxicity to clinical bacterial strains of *S. aureus,* including methicillin-resistant *S. aureus* [77].

This broad spectrum of activities against bacteria, fungi and plants suggests that the molecular targets of solanapyrones are key cellular components. Indeed, solanapyrone A is known to specifically bind to X-family DNA polymerases in vitro [80]. This particular DNA polymerase family exerts its function exclusively on DNA repair process and cell cycle control in the DNA replication processes during mitosis and meiosis [81,82]. Recently, increasing evidence suggests that DNA damage and subsequent repair processes are linked to the induction of plant immune responses [83,84,85,86]. Therefore, it is plausible that solanapyrone A inhibits DNA repair processes, induces cell cycle arrest, and then causes apoptosis. Alternatively, solanapyrone A could affect a defense signaling pathway induced by DNA damage and subsequent repair processes in plants.

### 2.6. Other Secondary Metabolites

**Figure 4:**
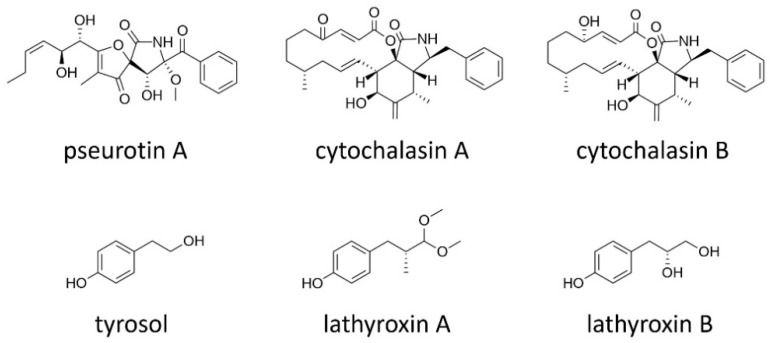
Secondary metabolites of hybrid polyketide–nonribosomal peptide origin and simple phenolics produced by *Ascochyta lentis*, *A. lathyri* and *A. lentis* var. *lathyri.*

Complex compounds of hybrid polyketide–nonribosomal peptide origin are also produced by *A. lentis* and *A*. *lathyri* (syn. *Boeremia exigua* var. *pseudolilacis*) (Figure 4). The heterospirocyclic compound pseurotin A that was identified in culture extracts of *A. lentis* exhibited mild phytotoxicity [40]. Pseurotin A is known as a potent inhibitor for chitin synthase [87] and as an anti-allergy agent, inhibiting immunoglobuline E production in vitro [88]. The well-known actin polymerization inhibitors cytochalasins A and B are produced by *A. lathyri* [89]. Cytochalasin D was also identified in the culture extract of an *A. rabiei* isolate [90]. However, the cytochalasin-producing isolate did not produce any of the solanapyrones, and was later found not to be *A. rabiei* (*personal communication*, S. S. Alam).

*Ascochyta lentis* and *A. lentis* var. *lathyri* produce simple phenolic compounds tyrosol and lathyroxins, respectively (Figure 4) [40,91]. Tyrosol was shown to be slightly phytotoxic [40]. Interestingly, tyrosol was identified as a quorum-sensing compound in *Candida albicans*, inducing filamentous growth and biofilm formation of this dimorphic fungus pathogenic to humans [92]. Lathyroxins A and B caused necrosis on plant leaves and inhibited seed germination of the parasitic weed *Phelipanche ramose* [91].

## 3. Biosynthesis Gene Cluster and Ecological Roles

### 3.1. Solanapyrone Biosynthesis Gene Cluster

The solanapyrone biosynthesis gene cluster comprises six genes (*sol1*–*sol6*), and was first identified in *Alternaria solani* [93]. Interestingly, the highly conserved gene cluster was also found in *A. rabiei*, showing overall 97% similarity at the amino acid sequence level [94]. The polyketide synthase (PKS) encoded by the *sol1* is classified as a highly-reducing PKS and is thought to be involved in biosynthesis of the precursor prosolanapyrone, an α-pyrone with an unsaturated alkyl chain. The production of such linear α-pyrones has been reported in *Al. solani* and related fungi [95,96,97,98], but the difference in solanapyrone biosynthesis from other linear α-pyrones is the involvement of a Diels–Alder reaction catalyzed by *sol5* to form a decalin ring from the unsaturated alkyl chain of prosolanapyrone [74,93,99].

As mentioned above, solanapyrones have been considered a pathogenicity factor due to their strong phytotoxicity [71,72]. However, genetic mutants of both *A. rabiei* and *Al. solani* lacking the Diels–Alderase enzyme lost the production of solanapyrone but remained pathogenic, suggesting that solanapyrones are not required for pathogenicity [74]. The expression patterns of the Diels–Alderase gene in *A. rabiei* also supported this observation, as the gene was specifically expressed at the site of sporulation, but not during plant infection [47]. Furthermore, solanapyrones were produced in *A. rabiei* growing on chickpea straw, and interestingly, xylan, when used as a sole carbon source, supported the greatest production of solanapyrones, suggesting the saprobic growth stage-specific production [47]. Similarly, brefeldin A was specifically produced during the saprobic growth of *P. medicaginis* [52]. Given the observed antimicrobial activities of solanapyrones produced by fungi occupying diverse ecological niches, solanapyrones may play a role in competition with other saprobic microbes.

### 3.2. Ascochitine Biosynthesis Gene Cluster

Ascochitine is structurally similar to the well-known mycotoxin citrinin produced by fungal species in the genera *Aspergillus*, *Monascus* and *Penicillium* [100,101]. A putative PKS gene for ascochitine biosynthesis was identified in *A. fabae* through a homology-based search with PKS genes responsible for citrinin biosynthesis, and its involvement in ascochitine biosynthesis was confirmed by gene knockout study [102]. Intriguingly, homologs of the ascochitine PKS gene were found in the genome sequences of multiple *A. lentis* isolates from which ascochitine was not detected [102]. Many single nucleotide polymorphisms were observed in the homologous PKS genes, which include nonsense mutations, resulting in no ascochitine production in *A. lentis* isolates [102].

The putative ascochitine gene cluster comprises eleven genes including a transcription factor (TF), a transporter, tailoring enzymes and proteins with unknown function [102]. The putative TF in the ascochitine gene cluster belongs to the fungal-specific Zn(II)2Cys6 zinc cluster TF family. However, it lacked a DNA-binding domain that is typically found in the TF family. Interestingly, this unusual type of TF was also found in the solanapyrone gene cluster, and it was reported that the TF encoded by the *sol4* positively regulates the whole solanapyrone gene cluster [94].

Pathogenicity tests with asochitine-minus mutants of *A. fabae* lacking the ascochitine PKS gene indicated that ascochitine was not essential for causing disease on faba bean. Unlike in another ascochitine-producing fungus *P. clematidina* [24], there was no positive correlation between the aggressiveness of *A. fabae* isolates and their capability in in vitro ascochitine production [103]. Ascochitine accumulated in aged hyphae where sporulation was initiated in *A. fabae* and *A. viciae-villosae*, suggesting ascochitine may have a role in combatting with microbial competitors in nature [102].

### 3.3. Ascofuranone Biosynthesis Gene Cluster

The transcriptome-based approach for identifying SM gene clusters was excellently applied to discover ascofuranone/ascochlorin biosynthesis gene cluster in *Acremonium egyptiacum* [104]. The ascofuranone/ascochlorin gene clusters were separated at two genomic loci, and the main gene cluster harboring a PKS gene was sufficient for ascochlorin biosynthesis [104]. Combining differential gene expression analyses and the functional prediction of genes by homology, the authors were able to locate the second gene cluster harboring three genes that are required for further modifications of an ascochlorin precursor to biosynthesize ascofuranone [104]. Although the complete biosynthetic pathway and responsible genes for the biosynthesis of ascofuranone/ascochlorin were identified, their ecological roles in the fungal biology remain to be determined.

### 3.4. Identification of Other SM Gene Clustesrs in the Legume-Associated Ascochyta Species

The advancement in bioinformatics tools and growing knowledge on PKS genes allowed the recent identification of ascochitine and ascofuranone/ascochlorin biosynthesis gene clusters [102,104]. The homology-based search and subsequent phylogenetic analysis of candidate PKS genes with known PKS genes that have been linked to biosynthetic pathways will aid in the future identification of SM gene clusters in the legume-associated *Ascochyta* and *Phoma* species.

A non-reducing PKS is required for the formation of the core anthraquinone structure in *Aspergillus nidulans* [105]. This information will help identify biosynthetic genes and associated gene clusters for anthraquinones produced by *A. lentis*. Also, a gene cluster including a highly-reducing PKS in *Eupenicillium brefeldianum* was proposed to be involved in the 13-membered macrolide brefeldin A [106]. In the genome of *A. pinodes*, a PKS gene similar to the brefeldin A PKS gene (38% DNA sequence identity) was found, suggesting its involvement in macrolide biosynthesis in *A. pinodes* [107]. Additionally, hybrid PKS–nonribosomal peptide synthetase (NRPS) genes required for pseurotin A and cytochalasins biosynthesis have been identified in *Aspergillus* spp. [108,109]. Homology-based searches for PKS and hybrid PKS–NRPS genes in the publicly available genome sequences of *A. lentis* and *A. pinodes* would provide an opportunity to identify SM gene clusters and study the involvement of phytotoxic SMs in pathogenicity of the legume-associated *Ascochyta* and *Phoma* species.

## 4. Conclusions

Many phytotoxic compounds have been isolated from cultures of *Ascochyta* and *Phoma* species, summarized in Table 1. The apparent phytotoxicity of the SMs produced by this fungal family has given us clues on the biological and ecological roles of SMs. To date, however, no SM produced by the legume-associated *Ascochyta* and *Phoma* species has been proven to be involved in fungal pathogenesis. The causal agents of the Ascochyta blight have been traditionally considered necrotrophs, producing necrotic lesions on the host plants and have been believed to kill or damage the host plants by secreting toxic compounds in order to thrive off dead or dying plant tissues. There remains a lack of knowledge on the biosynthetic genes for production of the phytotoxic compounds. To the best of our knowledge, only three SM gene clusters have been linked to chemically defined phytotoxic compounds in *Ascochyta* and *Phoma* species. With the advent of high-throughput sequencing technologies, fungal genomes are becoming exponentially available and it becomes clear that we just have scratched the surface in terms of the identification of SM gene clusters. Thus, many more await their discovery. Growing genomic resources and knowledge on PKS and other biosynthetic genes will facilitate the identification of SM gene clusters and enable us to test the hypotheses on the role of the phytotoxic compounds in pathogenicity.

## Figures and Tables

**Table 1:** Secondary metabolites produced by *Ascochyta* and *Phoma* species.

Compounds	Class	Biological Function	Producing Fungi (Host) [Reference]
ascochitine	polyketide	antifungal phytotoxic	*Ascochyta fabae* (faba bean) [16,23,102]; *A. hyalospora* (lambsquarters) [25]; *A. pisi* (pea) [16,22]; *A. salicorniae* (*Ulva* sp., an alga) [27]; *A. viciae-villosae* (hairy vetch) [16,102]; *Phoma clematidina* (*Clematis* spp.) [24]; *P. koolunga* (pea) [16]; many *Ascochyta*-like spp. isolated from different legumes [16]
ascochlorin	meroterpenoid	antitrypanosomal	*A. viciae* (*Vicia* spp., vetches) [56,64]
ascofuranone	meroterpenoid	antitrypanosomal	*A. viciae* (*Vicia* spp., vetches) [57,65]
ascosalitoxin	polyketide	phytotoxic	*A. salicorniae* (*Ulva* sp., an alga) [27]
brefeldin A	polyketide	phytotoxic	*P. medicaginis* (*Medicago* spp.) [52]
cytochalasin A/B	hybrid ^1^	cytotoxic	*A. lathyri* (grass pea) [89]
herbarumin II	polyketide	phytotoxic	*A. pinodes* (pea) [43,53]
lathyroxin A/B	simple phenolics	phytotoxic	*A. lentis* var. *lathyri* (grass pea) [91]
lentiquinone A	polyketide	antibacterial (mild) phytotoxic	*A. lentis* (lentil) [41]
lentiquinone C	polyketide	antibacterial phytotoxic (mild)	*A. lentis* (lentil) [41]
lentisone	polyketide	antibacterial, phytotoxic	*A. lentis* (lentil) [40]
pachybasin	polyketide	induction of mycoparasitic coiling	*A. lentis* (lentil) [43]
pinolidoxin	polyketide	phytotoxic	*A. pinodes* (pea) [16,42]; *A. pinodella* (chickpea, lentil) [16]
pseurotin A	hybrid ^1^	phytotoxic (mild)	*A. lentis* (lentil) [40]
putaminoxin	polyketide	phytotoxic	*A. pinodes* (pea) [43]
pyrenolide A	polyketide	antifungal	*A. hyalospora* (lambsquarters) [25]
solanapyrone A	polyketide	phytotoxic, antibacterial antifungal	*A. rabiei* (chickpea) [16,47,70]
solanapyrone C	polyketide	algistatic, antibacterial	*A. rabiei* (chickpea) [16,47,70]
tyrosol	simple phenolics	phytotoxic (mild) quorum sensing	*A. lentis* (lentil) [40,92]

^1^ polyketide–nonribosomal peptide hybrid origin.
